# What Will B Will B: Identifying Molecular Determinants of Diverse B-Cell Fate Decisions Through Systems Biology

**DOI:** 10.3389/fcell.2020.616592

**Published:** 2021-01-12

**Authors:** Simon Mitchell

**Affiliations:** Brighton and Sussex Medical School, University of Sussex, Brighton, United Kingdom

**Keywords:** systems biology, B-cells, computational modeling, heterogeneity, cell signaling, cell fate, NF-κB, cell-to-cell variability

## Abstract

B-cells are the poster child for cellular diversity and heterogeneity. The diverse repertoire of B lymphocytes, each expressing unique antigen receptors, provides broad protection against pathogens. However, B-cell diversity goes beyond unique antigen receptors. Side-stepping B-cell receptor (BCR) diversity through BCR-independent stimuli or engineered organisms with monoclonal BCRs still results in seemingly identical B-cells reaching a wide variety of fates in response to the same challenge. Identifying to what extent the molecular state of a B-cell determines its fate is key to gaining a predictive understanding of B-cells and consequently the ability to control them with targeted therapies. Signals received by B-cells through transmembrane receptors converge on intracellular molecular signaling networks, which control whether each B-cell divides, dies, or differentiates into a number of antibody-secreting distinct B-cell subtypes. The signaling networks that interpret these signals are well known to be susceptible to molecular variability and noise, providing a potential source of diversity in cell fate decisions. Iterative mathematical modeling and experimental studies have provided quantitative insight into how B-cells achieve distinct fates in response to pathogenic stimuli. Here, we review how systems biology modeling of B-cells, and the molecular signaling networks controlling their fates, is revealing the key determinants of cell-to-cell variability in B-cell destiny.

## Introduction

Following antigen exposure, B-cells are activated, often with the help of T-cells, to secrete antibodies essential for resolving infections. In addition to this well-studied humoral immune function, an important role for B-cells in cellular immunity is emerging (Hoffman et al., 2016). B-cell diversity is vital, with loss of diversity correlating with frailty and reductions in overall survival (Gibson et al., 2009). Each B-cell’s destiny can range from apoptosis within hours, rapid differentiation into a short-lived plasma blast in the initial days of an infection (Lam and Baumgarth, 2019), to that of a memory B-cell surviving for decades (Seifert and Küppers, 2016), or long-lived plasma cells found in the bone marrow > 40 years after vaccination (Brynjolfsson et al., 2017). Even B-cells stimulated *ex vivo*, without the complexities of T-cells and the germinal center, will undergo varied fates (Hawkins et al., 2009; Mitchell et al., 2018). Single-cell measurements of B-cells, stimulated with B-cell receptor (BCR)-independent stimuli, show vast cell-to-cell heterogeneity (Shih et al., 2002; Hawkins et al., 2013). Therefore, it seems that non-genetic B-cell diversity is an intrinsic property of B-cells. This has led to substantial efforts to identify the molecular determinants of B-cell destiny with pivotal studies combining insight from experimental models with *in silico* systems biology models. We will first discuss molecular determinants of each fate decision in isolation, followed by the molecular signaling pathways that interpret the cell’s environment. Finally, we will put the pieces together to describe how cell-to-cell variability in B-cell fates is understood through systems biology.

## Cell Cycle

In response to antigen challenge, the B-cell population expands due to a portion of the cell population undergoing repeated rounds of cell division. *In vitro*, between 0 and 8 divisions occur, while multiple rounds of proliferation in the germinal center can lead to substantially higher (30+) divisions (Duffy et al., 2012; Tas et al., 2016; Mitchell and Hoffmann, 2018).

Mathematical models have been central to studies of the cell cycle since the 1960s, starting with phenomenological models recapitulating cell-cycle phase transitions (Smith and Martin, 1973). Dowling et al. (2014) observed that time spent in both G1 and S/G2/M phases is highly variable in B-cells. As a result, they proposed an alternative to the highly influential Smith–Martin model, in which all phases of the cell cycle stretch depending on a stochastically determined total division time (Smith and Martin, 1973). The timing of cell-cycle phases was found to be highly correlated in sister cells, suggesting a pre-existing non-genetic source of variability strongly inherited through cell division (Dowling et al., 2014). Interestingly, this stretching of all cell-cycle phases proportional to total cell-cycle length does not seem to be maintained in B lymphoma cell lines (Pham et al., 2018). An inherited molecular source of cell-to-cell variability is consistent with results from lineage-tracking of division times across multiple generations in proliferating B lymphocytes (Duffy et al., 2012; Mitchell et al., 2018). Heinzel et al. (2017) identified c-Myc as this molecule and fit a mathematical model to experimental data based on distributed c-Myc controlling a distributed division destiny. B-cell-specific modeling of cell division has been restricted to phenomenological modeling without explicitly representing molecular processes (Callard and Hodgkin, 2007; Zilman et al., 2010).

**FIGURE 1:**
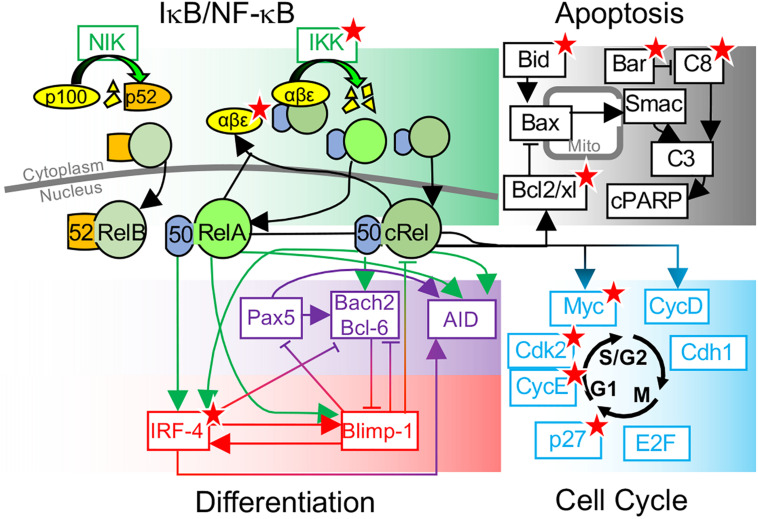
Overview of key molecular determinants of B-cell fate decisions. Schematic of B-cell fate decisions determined by molecular networks controlling NF-κB, Apoptosis, differentiation, and the cell cycle. Key molecular determinants of cell-to-cell variability in B-cell fate decisions, identified through systems biology modeling and experimental studies, are indicated (★).

Kinetic modeling of the eukaryotic cell cycle became possible as increasing molecular mechanistic detail was revealed in the 1990s (Novak and Tyson, 1993; Csikasz-Nagy, 2009). The foundations for this progress was provided by the seminal work of Novák and Tyson, whose models have a striking ability to generate predictions validated many years later by experiments (Pomerening et al., 2003; Sha et al., 2003; Novák and Tyson, 2004). By adapting metabolic control analysis approaches to this model of the cell cycle, Conradie et al. (2010) found that variation in Cdk2 and its interactions with cyclin-dependent kinase inhibitor (p27^Kip1^) and CyclinE were the most likely sources of cell-to-cell variability in the cell cycle. Later, live-cell Cdk2 tracking identified a bifurcation in Cdk2 trajectories, controlled by p27, as a source of cell-to-cell heterogeneity (Spencer et al., 2013; Figure 1). Despite the fact that much of this mechanistic insight has been generated from models of non-lymphatic cell lines, the ability of mechanistic cell-cycle models to generate insights into multiple model organisms from yeast to xenopus suggests that the molecular architecture of such models can also be informative in B-cells (Pomerening et al., 2005; Skotheim et al., 2008). Indeed, a generic model of the mammalian cell cycle was incorporated into a multiscale B-cell model by Shokhirev et al. (2015), which replicated single-cell B-cell proliferation measured by time-lapse microscopy.

## Cell Death

Mounting an antibody response requires a balance of B-cell proliferation and cell death. Inadequate apoptosis leads to auto-immunity and malignancies while excess apoptosis can cause immunodeficiency (Cossu, 2010; Correia et al., 2015; Li et al., 2016; Yang et al., 2016). Mathematical models have been widely used to illuminate the cell-to-cell variation in the timing of apoptosis (Spencer and Sorger, 2011). The first kinetic models of apoptosis were published two decades ago (Fussenegger et al., 2000), as single-cell analysis revealed switch-like effector caspase kinetics in individual cells (Goldstein et al., 2000; Tyas et al., 2000). This switch-like behavior motivated construction of computational models, with multiple mechanisms being proposed from receptor clustering to cooperativity in apoptosome formation or pore formation (Eissing et al., 2004; Hua et al., 2005; Bagci et al., 2006; Legewie et al., 2006; Ho and Harrington, 2010). Comprehensive mechanistic models constructed by Peter Sorger’s group, combined with single-cell fate tracking, have been instrumental in understanding cell-to-cell variability in apoptosis (Albeck et al., 2008; Spencer et al., 2009). These studies attributed variability in cell death timings to differences in BID truncation and a threshold determined by the Bcl-2 family proteins (Spencer et al., 2009; Figure 1). Apoptosis timing was found to be correlated in recently divided cells, but correlations between sister cells were lost quickly following cell division (*t*_1__/__2_ = 1.5 h) due to intrinsic gene expression noise (Spencer et al., 2009). Similar analysis in B-cells found similar transient correlations in sibling cell apoptosis timing (Hawkins et al., 2009). This may seem inconsistent with results showing that, in proliferating B-cells, the majority of cells that are progeny of a single founder cell will undergo apoptosis in the same generation, even following 100+ hours of proliferation (Hawkins et al., 2009; Mitchell et al., 2018). It seems that the cell-to-cell variability in the decision to undergo apoptosis in a particular generation, and the precise timing of apoptosis, have distinct sources. This is consistent with an analysis of the Albeck et al. (2008) model performed by Loriaux et al. (2013), which found that molecular determinants of the timing of apoptosis are not equivalent to molecular determinants of whether or not a cell undergoes apoptosis. This analysis suggests that Procaspase 8 and its negative regulator Bar are key determinants of cell-to-cell differences in apoptotic decision making (Loriaux et al., 2013). Recent Luria–Delbrück analysis of gene expression revealed that the set of genes whose expression is reliably inherited differs between cell types (Luria and Delbrück, 1943; Shaffer et al., 2020). Therefore, key to predictive mechanistic modeling of B-cell apoptosis will be understanding the molecular determinants of apoptotic decisions, along with how reliably these factors are inherited during proliferation.

## B-Cell Differentiation

Following proliferation, activated B-cells differentiate into short-lived plasma blasts or long-lived plasma cells, both of which are antibody-secreting cells (ASCs) (Shapiro-Shelef and Calame, 2005). Cell division is required but not sufficient for ASC production, and considerable cell-to-cell differences in the timing of differentiation following activation exist, even *in vitro* (Tangye and Hodgkin, 2004; Zhou et al., 2018).

Recent single-cell RNAseq data indicate a bifurcation during the early stages of B-cell activation, committing a portion of cells to an ASC destiny (Scharer et al., 2020). This requires Interferon Regulatory Factor 4 (IRF4) induction, with higher and sustained activation biasing cells toward ASC fates (Ochiai et al., 2013). This was also seen by Xu et al. (2015) who constructed a minimal mathematical model of mutual inhibition between IRF4 and IRF8 in B-cells, with initial conditions obtained by flow cytometry, and found bifurcating fates recreating experiments showing a fraction of cells undergo rapid differentiation into plasma blasts. Sciammas et al. (2011) modeled the core regulatory network controlling terminal differentiation of activated B-cells including the mutual inhibition between Blimp1 and Bcl6/Bach2, along with the incoherent effects of IRF4 activating both somatic hypermutation (through AID) and differentiation (through Blimp1). This molecular model was incorporated into multiscale stochastic simulations, which revealed that differences in the time spent undergoing class-switch recombination and somatic hypermutation could be explained by the initial rate of IRF4 activation (Sciammas et al., 2011). Subsequent kinetic modeling found that interactions between Irf4, Bcl6, and Blimp1 were sufficient to capture a broad variety of B-cell differentiation dynamics (Martínez et al., 2012). Taken together, these results show that cell-to-cell differences in terminal differentiation of B-cells result from differences in IRF4 signaling.

## NF-κB

NF-κB is a dimeric transcription factor, first discovered in B-cells and later revealed to have near-ubiquitous expression (Sen and Baltimore, 1986; William et al., 1995; Xu et al., 1996; Inlay et al., 2002; Baltimore, 2009). NF-κB’s important role in B-cell development, survival, and function has been widely studied (Vallabhapurapu and Karin, 2009; Gerondakis and Siebenlist, 2010; Kaileh and Sen, 2012; Heise et al., 2014; Almaden et al., 2016). In response to increasing BCR activation, B-cells show a digital all-or-nothing NF-κB response, with an increasing number of cells responding, rather than each cell increasing its response, with increasing NF-κB (Shinohara et al., 2014). The all-or-nothing response suggests the presence of a positive feedback loop, enabling cells that cross a cell-specific threshold of activation to invariably achieve maximum activation. Through iterative computational and experimental modeling, a positive feedback was identified between TAK1 (MAP3K7) and inhibitor of NF-κB (IκB) kinase-β (IKKβ) complex, resulting in switch-like single-cell behaviors; disruption of this feedback results in a more graded response (Shinohara et al., 2014). These all-or-nothing responses are consistent with studies applying information theoretic approaches to NF-κB signaling, which reveal that intrinsic noise in NF-κB limits the information the pathway can encode about each cell’s environment to only a few states, e.g., absence, low and high stimuli (Cheong et al., 2011; Selimkhanov et al., 2014; Mitchell and Hoffmann, 2018). It seems unlikely that the intricate environmental stimuli received by B-cells through diverse receptors can be accurately encoded through noisy NF-κB signaling in single cells (Rawlings et al., 2012). This may be reconciled by a model-aided analysis that revealed a trade-off between reliable single-cell responses and reliable population-scale responses, with distributed switch-like responses enabling an appropriate fraction of cells within a population to reliably respond (Suderman et al., 2017).

Core to NF-κB signaling is its regulation through sequestration in the cytoplasm by inhibitory proteins (IκBs) (Mitchell et al., 2016). IκBs are themselves induced by nuclear NF-κB, resulting in a negative feedback in which NF-κB inhibits itself with a delay due to gene expression and protein synthesis (Figure 1). Such systems can create the oscillatory dynamics seen in NF-κB signaling, and mathematical modeling has been central to understanding NF-κB (Hoffmann et al., 2002; Lipniacki et al., 2004; Nelson et al., 2004; Basak et al., 2012). Each IκB family member has distinct kinetics of induction, degradation, and NF-κB sequestration, resulting in distinct contributions to cell-to-cell variability. IκBα displays rapid and robust stimulus-dependent degradation and subsequent NF-κB-dependent induction, creating a noise-insensitive first peak of NF-κB activity. IκBε has slower kinetics than IκBα (Kearns et al., 2006). Incorporating IκBε with slower negative feedback into mathematical simulations revealed that IκBε enables a more reliable dose-dependent response to sustained signals, minimizing the impact of stochastic gene expression on late-phase NF-κB activity (Longo et al., 2013). Through both kinetic modeling and experimental investigation, IκBε has been found to limit B-cell expansion through limiting NF-κB cRel and RelA (Alves et al., 2014).

Whether cell-to-cell differences in NF-κB signaling result from intrinsically generated noise, such as transcriptional noise, or pre-existing differences between B-cells prior to stimulation has been debated (Williams et al., 2014). Both sources of variation have been simulated through mathematical modeling of NF-κB, with intrinsic noise recreated through stochastic simulation using the Gillespie algorithm (Gillespie, 1977) and pre-existing variability simulated by sampling parameters prior to deterministic simulations (Hayot and Jayaprakash, 2006; Cheng et al., 2015; Hughey et al., 2015). Recent studies combining mathematical modeling with single-cell analysis find that pre-existing cell-to-cell differences best explain distributed single-cell NF-κB dynamics and the similar responses observed in daughter cells (Cheng et al., 2015; Hughey et al., 2015).

## Putting the Pieces Together

In studying the regulatory networks controlling the B-cell fate decisions described above, a pattern emerges. Key molecular determinants of cell-to-cell variability in B-cell fate decisions are NF-κB target genes. Indeed, recent single-cell RNA-sequencing analysis found that the most highly variable genes in lymphoid cells were functionally significant and centered around NF-κB and its target genes, including NFKBIA, MYC, IRF4, and AID (Osorio et al., 2020).

Myc and Bcl2 are NF-κB target genes that have been shown to control B-cell division and apoptosis (Duyao et al., 1990; Chen et al., 1999; Catz and Johnson, 2001; Figure 1). This was used by Shokhirev et al. (2015) in order to connect models of NF-κB signaling, the cell cycle, and apoptosis networks discussed above, recapitulating cellular statistics from single-cell time-lapse microscopy and revealing that NF-κB cRel was essential to protect growing B-cells from apoptosis. Mitchell et al. (2018) used this model to determine the source of cell-to-cell variability using single-cell lineage tracking experiments and discovered that B-cell fates were determined by molecular differences in the naïve B-cell population that are reliably inherited during proliferation. Interestingly, predictions of the most significant molecular determinants of cell-to-cell fate variability depend on the magnitude of variability. Perturbing parameters controlling NF-κB signaling resulted in the largest changes in B-cell proliferation; however, this required relatively large parameter changes of twofold or more. Smaller changes in parameters, and logistic regression on simulated cell populations with experimentally determined molecular heterogeneity, did not identify NF-κB-related biochemical processes as the largest determinants of cell-to-cell variability in B-cell proliferation. Instead, apoptotic signaling regulators such as Bar, Caspase 3, and XIAP were predicted to be the most significant determinants of B-cell proliferative outcome, a result tested through caspase inhibition (Mitchell et al., 2018).

**FIGURE 2:**
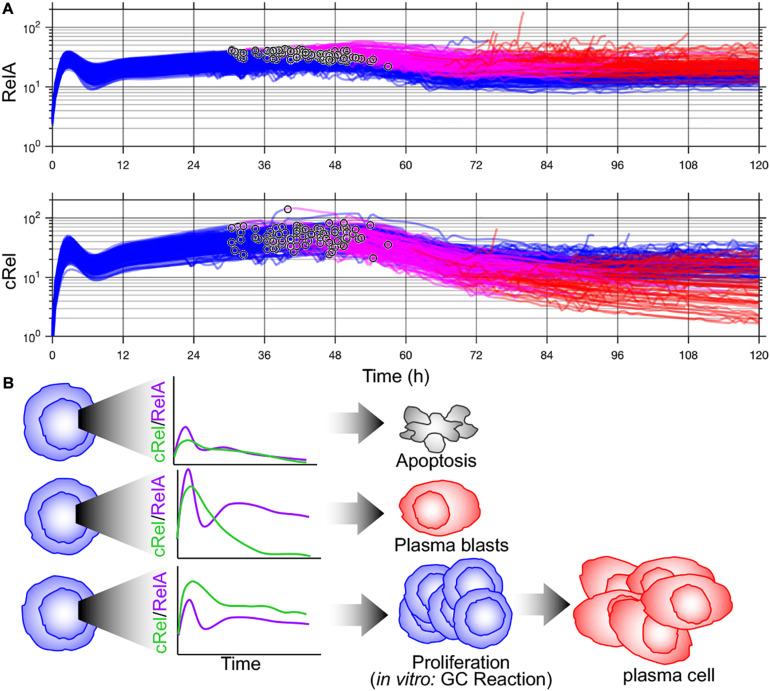
Multiscale modeling of B-cell fates predicts cell-to-cell variability in NF-κB subunits that can orchestrate distinct fates. **(A)** Time course of NF-κB RelA (top) and NF-κB cRel (bottom) from multiscale simulation data from Mitchell et al. (2018). Blimp-1 upregulation time is indicated (°). Activated B-cells (blue), high Blimp1 (pink), and high Blimp1 with low AID (complete differentiation, red). **(B)** Proposed orchestration of cell fates through NF-κB. Inadequate NF-κB induction results in apoptosis (top). High RelA induction followed and subsequent dynamic cRel downregulation results in rapid plasma blast differentiation. High cRel with lower RelA (and therefore lower IRF4) results in a proliferative/germinal center phenotype.

Key determinants of cell-to-cell variability in B-cell terminal differentiation including Blimp1 and IRF4 are also NF-κB target genes (Grumont and Gerondakis, 2000; Morgan et al., 2009; Heise et al., 2014). This led Roy et al. (2019) to add NF-κB regulation to the model of Sciammas et al. (2011) and discover that a previously unidentified regulatory interaction was required to recapitulate experimental results. Roy et al. (2019) discovered that the missing interaction was transcriptional inhibition of NF-κB cRel by Blimp1 and that dynamic downregulation of cRel by Blimp1 was required for plasma cell differentiation (Roy et al., 2019). Once this new regulatory interaction was incorporated into the multiscale model of Shokhirev et al. (2015), the model recapitulated cell-to-cell variability in B-cell proliferation and differentiation dynamics from wild-type and knockout mice (Roy et al., 2019). Given the overlap between NF-κB target genes and key determinants of B-cell fate decisions, well characterized cell-to-cell variability in NF-κB may coordinate diverse B-cell fates. Indeed, if Blimp1 upregulation time is noted in simulations from Roy et al. (2019), this model predicts that B-cells with the highest NF-κB RelA differentiate more quickly (Figure 2). As NF-κB integrates BCR and toll-like receptor signaling and induces IRF4, this prediction is consistent with the rapid differentiation by high-affinity BCR-expressing B-cells into plasma blasts (Paus et al., 2006) and the rapidly differentiating subset of cells with high IRF4 activation (Xu et al., 2015). Subsequent cRel downregulation is required to complete differentiation (Roy et al., 2019). The distinct roles of NF-κB cRel and RelA in B-cell survival and differentiation, respectively, seen in these multiscale models are consistent with *in vivo* requirements for germinal center maintenance and plasma cell generation (Heise et al., 2014) and an emerging picture of subunit-specific dysregulation of NF-κB in lymphoid malignancies (Kennedy and Klein, 2018).

## Discussion

The decision each B-cell faces, between proliferation, apoptosis, and differentiation, is vitally important to thread the needle between autoimmunity and immunodeficiency. An effective immune response requires a portion of B-cells to rapidly express antibodies, in order to buy time for the germinal center reaction to iteratively refine and expand an antigen-specific B-cell population to resolve the infection. Not only does this require careful coordination of multiple cell fates within each B-cell, but it requires cells to reach distinct decisions to the same challenge. Mathematical modeling provides unique opportunities to quantitatively disentangle the cell-intrinsic and extrinsic sources of cell-to-cell variability. Through combined modeling and single-cell experiments, we now know that distinct B-cell fates are achieved through molecular differences in the founder cell of each lineage, which are reliably inherited across many rounds of cell division (Hawkins et al., 2009; Mitchell et al., 2018).

B-cell differentiation takes place in the germinal centers of the spleen and lymph nodes. These structures spatially organize and traffic B-cells, enabling interactions with antigen-presenting cells and T-cells (De Silva and Klein, 2015; Mesin et al., 2016). Recently, these extra- and intercellular processes have been modeled through stochastic approaches (Thomas et al., 2019; Pélissier et al., 2020). Integrating the molecular determinants of B-cell fate decision into models of B-cell fates within the germinal center will be informative for therapeutic targeting of B-cells (Kepler and Perelson, 1993; Figge, 2005; Meyer-Hermann et al., 2012; Robert et al., 2017; Thomas et al., 2019; Pélissier et al., 2020; Verheijen et al., 2020).

The ultimate goal of many of the studies discussed here, and systems biology as a whole, is to use models to enable predictive control over cells in health and disease. While the emerging picture from experiments and models is that measuring one or even 5+ (Mitchell et al., 2018) molecular abundances is unlikely to reliably predict a B-cell’s fate, this does not preclude reliable interventions. Modeling has identified molecular targets to control B-cell fates and predicted how mutations will skew proportions and timings of cell fate decisions in experimental systems (Mitchell et al., 2018; Roy et al., 2019). One challenge to predictive modeling is that many models have been parameterized in other cell types, and B-cell specific parameterization is daunting. However, the prevalence of single-cell data, along with promising model-generated experiment-validated results, suggests that a systems biology approach to predictably controlling B-cell responses is a realistic goal.

## Author Contributions

SM conceived the study and wrote the manuscript.

## Conflict of Interest

The author declares that the research was conducted in the absence of any commercial or financial relationships that could be construed as a potential conflict of interest.
